# Gitelman's syndrome with persistent hypokalemia - don't forget licorice, alcohol, lemon juice, iced tea and salt depletion: a case report

**DOI:** 10.1186/1752-1947-5-312

**Published:** 2011-07-14

**Authors:** Urs Knobel, Goli Modarres, Markus Schneemann, Christoph Schmid

**Affiliations:** 1 Endocrinology, Diabetology and Clinical Nutrition, Department of Internal Medicine, University Hospital of Zürich, Rämistrasse 100, CH-8091 Zürich, Switzerland; 2 Internal Medicine, Department of Internal Medicine, University Hospital of Zürich, Rämistrasse 100, CH-8091 Zürich, Switzerland

## Abstract

**Introduction:**

Chronic hypokalemia is the main finding in patients with Gitelman's syndrome. Exogenous factors can trigger deterioration of the patient's condition and provoke clinical symptoms. We discuss the pathophysiology of and therapy for Gitelman's syndrome, with a focus on dietary factors which may aggravate the disease.

**Case presentation:**

We describe the case of a 31-year-old, previously apparently healthy Caucasian Swiss man who presented to our hospital with gait disturbance of subacute onset and a potassium level of 1.5 mmol/L. A detailed medical history revealed that he had been consuming large amounts of licorice (in the form of Fisherman's Friend menthol eucalyptus lozenges). Despite discontinuing the intake of glycyrrhizinic acid, his potassium level remained low. Biochemical investigations showed refractory hypokalemia and secondary hyperaldosteronism, suggestive of Gitelman's syndrome. Despite treatment with supplementation of potassium and magnesium in combination with an aldosterone antagonist, further clinically symptomatic episodes occurred. Triggers could be identified only by repeated detailed history taking. In response to the patient's dietary excesses (ingestion of relevant amounts of alcohol, lemon juice and iced tea), his hypokalemia was aggravated and provoked clinical symptoms. Finally, vomiting and failure to replace salt led to volume depletion and hypokalemic crisis, with a plasma potassium level of 1.0 mmol/L and paralysis with respiratory failure necessitating not only infusion of saline and potassium but also temporary mechanical ventilation.

**Conclusion:**

Dietary preferences may have a much larger impact than any drug treatment on the symptoms of this chronic syndrome. Individual (mainly dietary) preferences must be monitored closely, and patients should be given dietary advice to avoid recurrent aggravation of hypokalemia with muscular weakness.

## Introduction

Hypokalemia is a common clinical problem. It can result from reduced potassium intake, increased translocation from extracellular spaces into the cells (as a transient condition) or, most commonly, from increased gastrointestinal or urinary losses. Increased potassium secretion in the distal nephron may account for such losses, for example, with the intake of diuretics or because of mineralocorticoid excess.

Clinically, a remarkable absence of arterial hypertension and occasional symptoms of hypokalemia, together with a biochemical constellation of persistent, refractory hypokalemia, metabolic alkalosis, secondary hyperaldosteronism, mild hypomagnesemia and hypocalciuria, are suggestive of Gitelman's syndrome. This autosomal recessive inheritable renal syndrome is caused by defective sodium chloride (NaCl) transporters in the distal convoluted tubule. In this case report, we discuss how exogenous factors such as licorice, alcohol, lemon juice, iced tea and low salt intake can further aggravate hypokalemia and provoke clinical symptoms.

## Case presentation

A 31-year-old, previously healthy Caucasian Swiss man was admitted to our hospital because of progressive weakness of the legs and muscle cramping which had developed over the course of a few hours. His clinical examination revealed paraparesis without sensory loss. Otherwise, the physical findings were unremarkable; his blood pressure in particular was within the normal range (130/80 mmHg). Biochemical analysis showed severe hypokalemia (1.5 mmol/L; normal range, 3.6 to 4.5 mmol/L), hypophosphatemia (0.42 mmol/L; normal range, 0.87 to 1.45 mmol/L) and mild hypomagnesemia (0.65 mmol/L; normal, 0.7 to 1.1 mmol/L). His calcium level was initially moderately elevated (with normoalbuminemia). His electrocardiogram (ECG) showed no arrhythmias, but changes characteristic of hypokalemia with increased amplitude of the U-wave were observed. His creatine kinase (CK) level was elevated (3425 U/L; normal value, < 190 U/L), indicating rhabdomyolysis. His renal function was moderately impaired (plasma creatinine, 110 μmol/L; normal range, 70 to 105 μmol/L). There was no evidence of hyperthyroidism (thyroid-stimulating hormone level, 2.3 mU/L; normal range, 0.27 to 4.2 mU/L). His level of active renin (measured on his second day in the hospital) was slightly elevated (90.5 mU/L; normal range, 5 to 50 mU/L), and his aldosterone level was within the normal range (117.2 ng/L; normal range, 30 to 200 ng/L). His urine had a low density of 1.010 g/mL (normal range, 1.020 to 1.030 g/mL), pH8.0, and contained traces of protein. The initial arterial blood gas analysis showed slight respiratory alkalosis (pH7.47, partial pressure of CO_2_, 4.39 kPa; partial pressure of O_2_, 8.91 kPa; HCO_3_, 24 mmol/L and base excess 0.7 mmol/L). His initial family history was non-contributory.

The patient ate a regular European diet without alcohol abuse and took no drugs (apart from smoking cigarettes). He had consumed roughly one and a half packets of original Fisherman's Friend menthol eucalyptus lozenges (Lofthouse of Fleetwood Ltd., Maritime Street, Fleetwood Lancs, UK) daily for the past three months, which corresponds to an intake of 60 to 90 mg/day of glycyrrhizinic acid.

The patient was initially treated with parenteral and enteral potassium, spironolactone und indomethacin. Within 24 hours, his potassium level began to rise and his neurological symptoms disappeared. After three days, the patient was asymptomatic; at this point, his potassium level had increased to 2.9 mmol/L (normal range, 3.6 to 4.5 mmol/L).

At the first follow-up visit two months after he was discharged from our hospital (while taking aldactone 100 mg, no potassium or magnesium supplements and having consumed no licorice), the patient still had intermittent cramping without signs of paresis. His potassium level had again dropped to 2.6 mmol/L (normal range, 3.6 to 4.5 mmol/L), and his magnesium level was low (0.56 mmol/L; normal range, 0.7 to 1.1 mmol/L). Repeat urinalysis showed increased urinary loss of potassium and magnesium with very low calcium excretion. His secondary hyperaldosteronism was clearly more pronounced than at the time he first presented to our hospital, with elevated levels of active renin (287.8 mU/L; normal range, 5 to 50 mU/L) and aldosterone (917 ng/L; normal range, 30 to 200 ng/L) in blood testing. Morning cortisol and urinary cortisol excretion were within the normal range.

Under these circumstances, we considered a tubulopathy causing renal potassium loss. The biochemical constellation of normal renal function, hypokalemia and mild hypomagnesemia was suggestive of Gitelman's syndrome.

We continued treatment with a low dose of an aldosterone antagonist (first spironolactone, later epleronone 25 to 50 mg/day), the highest tolerated dose of magnesium (10 mmol daily) and potassium supplements. On this therapeutic regimen, stable hypokalemia (at around 2 mmol/L) could be established.

As an outpatient, our patient reported a few episodes of muscle weakness and gait disturbance which followed nights with moderately severe alcohol consumption. After these episodes disappeared when the patient refrained from drinking large amounts of alcohol, more severe hypokalemia developed a few months later. The patient admitted that he had begun drinking large amounts of lemon juice, in the form of either seven or eight freshly prepared lemons daily or by consuming a total of 1 L of pure lemon juice daily. Another episode of symptomatic hypokalemia requiring admission to our hospital as an in-patient was poorly understood but later history taking revealed that the patient, having experienced weakness, thirst and moderate weight loss, decided to replace fluid by drinking, for two days, 5 to 7 L of fluid daily, of which 4 L were consumed in the form of sugar-containing iced tea. This time muscle weakness had progressed from his legs to his upper extremities. Apart from hypokalemia (1.2 mmol/L), hypophosphatemia was more pronounced (0.26 mmol/L) despite a moderate rise in creatinine (to 158 μmol/L). His sodium level was 132 mmol/L, and his fasting glucose level was somewhat elevated (8.6 mmol/L).

The patient had progressive truncal weakness and finally presented with respiratory failure, necessitating temporary mechanical ventilation. At this point, extreme hypokalemia of 1.0 mmol/L was evident; his creatinine level was 206 μmol/L. For reasons of his own, the patient had started a new diet with restricted sodium intake. Two days after being weaned from assisted ventilation, the patient insisted on being discharged from our hospital. Four days later he had a potassium level of 2.6 mmol/L and a normal resting ECG reading, and a prolonged QTc time was found only during a bicycle exercise test, during which he showed a normal exercise performance of up to 150 W.

## Discussion

Our patient, a 31-year old, previously healthy Caucasian Swiss man, had a case of impressive symptomatic hypokalemia. His neurological symptoms (cramping and muscle weakness) resolved rapidly after correction of hypokalemia.

Hypokalemic paresis (paralysis) may be acquired in patients with thyrotoxicosis [1]. Our patient showed neither clinical nor biochemical signs of this disease, which is mainly found in Asians but still is more common as a cause of severe neurological symptoms in a patient presenting with hypokalemia in our hospital than Gitelman's syndrome, with the latter being more commonly found by chance on the basis of a laboratory finding of low potassium. Our patient had no history suggestive of familial periodic paralysis. This rare, hereditary defect of calcium or magnesium channels in skeletal muscles enhances the likelihood that insulin secreted after the intake of carbohydrate-rich food or catecholamine bursts (in response to stress or exertion) will result in increased potassium entry into the muscle, so that a substantial decrease in plasma potassium levels can ensue.

In our patient, we suspected a connection between his hypokalemia and his prolonged intake of Fisherman's Friend lozenges. These sweets contain licorice and the active substance glycyrrhizinic acid, which inhibits the renal enzyme 11β-hydroxysteroid dehydrogenase (type 2) and thereby converts cortisol (with mineralocorticoid activity) into cortisone (without mineralocorticoid activity). The local (paracrine) renal cortisol excess then leads to heightened mineralocorticoid receptor-mediated effects (pseudohyperaldosteronism or a syndrome of apparent mineralocorticoid excess). Regulated by intact adrenocorticotropic hormone feedback, systemic cortisol levels remain stable. Regulated by intact renin-angiotensin II loop feedback, renin activity ought to be suppressed. In a healthy individual, glycyrrhizinic acid (above 50 mg daily)-induced pseudohyperaldosteronism may cause hypertension within 14 days [2]. Furthermore, chronic ingestion will lead to hypokalemia with its known symptoms (muscle weakness, muscle pain and palsy).

Making the correct diagnosis depends on obtaining a precise medical history. Biochemical analysis shows the typical constellation of exogenously triggered excessive mineralocorticoid activity with suppressed renin activity, low aldosterone levels and metabolic alkalosis.

In patients in whom it is unclear whether licorice has been ingested, 24-hour urine collection may be helpful. The atypical constellation of a free cortisone level lower than the free cortisol level (reduced 11β-hydroxysteroid dehydrogenase activity) should raise clinical suspicions of pseudohyperaldosteronism [3].

Our patient presented with hypokalemia, but without hypertension. Despite having discontinued his consumption Fisherman's Friend lozenges, his hypokalemia persisted and his potassium remained at levels of around 2 mmol/L. Although they were checked only on the second day of his hospital stay, his active renin and aldosterone did not appear to be fully suppressed, but were rather slightly increased and normal, respectively. In the later course of his illness, these values increased further, demonstrating more clearly the secondary hyperaldosteronism characteristic of his chronic disorder. This presentation implied that the patient had experienced previous chronic hypokalemia with glycyrrhizinic acid-triggered decompensation. It may be rather unique (but fully compatible with his underlying disorder) that increased cortisol activity at the mineralocorticoid receptor could not fully suppress renin and did not induce arterial hypertension in this patient.

In normotensive patients with secondary hyperaldosteronism, not only diuretic use or abuse but also tubulopathy causing renal potassium loss should be considered. Normal renal function, hypokalemia, hypermagnesiuria with hypomagnesemia and (in not documented in our patient) metabolic alkalosis are diagnostic for Gitelman's syndrome, a disease mostly detected in young adults [4].

As discussed in the review article by Knoers and Levtchenko [5], this autosomal recessive disease (OMIM 263800) is the most frequent inherited salt-losing renal tubulopathy (prevalence estimated at 1:40,000). In the great majority of patients, it is caused by one of more than 140 described mutations in the solute carrier family 12, *SLC12A3 *gene (chromosome 16q13), encoding the renal thiazide-sensitive NaCl co-transporters (NCCT), expressed in the apical membrane of the first part of the distal convoluted tubule. An inherited origin of our patient's disease was initially far from obvious, and, up to the time of this writing, genetic testing has not been performed so that the mutations underlying his disorder remain uncharacterized. Most of the patients are compound heterozygous with different NCCT mutations on each allele [6]. Several years after our patient's initial presentation to our hospital, we learned from his sister, who was visiting him in the intensive care unit, that she also was found to have hypokalemia ranging between 2.6 and 3 mmol/L, but that she had never had any symptoms of the disorder.

Impaired NaCl reabsorption results in mild volume depletion. Secondary hyperreninemic hyperaldosteronism tends to correct sodium homeostasis at the expense of depletion of potassium and hydrogen ions, resulting in hypokalemia and metabolic alkalosis.

Elevated magnesium excretion in the proximal tubule is assumed to cause hypomagnesemia. Occasionally, associated chondrocalcinosis is probably caused by chronic hypomagnesemia. The pathogenesis of hypocalciuria is not entirely clear, but may be due either to extracellular volume contraction and increased passive Calcium reabsorption in the proximal tubule or to reduced luminal NaCl entry into distal convoluted tubular cells, cellular hyperpolarization and a basolateral calcium shift. Hypophosphatemia is not regularly observed. Apart from tubular dysfunction (with reduced phosphate reabsorption), metabolic alkalosis can increase phosphate excretion, and the accompanying hypokalemia further increases renal phosphate clearance [7].

The differential diagnosis includes classical Bartter's syndrome (especially type III). In classic Bartter's syndrome, patients typically present with severe symptoms (polyuria, polydipsia, dehydration and vomiting) in early childhood and often exhibit dysmorphic syndromes. Retardation of growth or mental development is a feature of untreated Bartter's syndrome. Different subtypes can be distinguished. All of them are characterized biochemically by hypokalemic metabolic alkalosis, but in contrast to the hypocalciuria in Gitelman's syndrome, Bartter's syndrome is characterized by hypercalciuria and medullary nephrocalcinosis is frequently observed [8].

Therapeutic options in Gitelman's syndrome are limited. As the tubulopathy cannot be modified, supplementation of potassium and magnesium is, apart from avoiding sodium depletion, the therapeutic cornerstone. However, the typically high substitution doses are associated with partly unacceptable gastrointestinal side effects. Thus, asymptomatic stable hypokalemia and borderline hypomagnesemia are a realistic therapeutic goal. Sufficient salt intake is important because low NaCl supply exaggerates volume depletion and increases the compensatory secondary hyperaldosteronism, resulting in intensified hypokalemia.

Although the use of an aldosterone antagonist can stabilize the hypokalemia, it competes with the compensatory secondary hyperaldosteronism. This can result in hypovolemia, possibly accentuated hyponatremia and eventually symptomatic hypotonia. Similar effects occur with the use of angiotensin-converting enzyme inhibitors or angiotensin 2 receptor blockers. Apart from isolated cases in which renal insufficiency developed, Gitelman's syndrome usually carries a very good prognosis.

Our patient was treated with a low dose of an aldosterone antagonist, the highest tolerated dose of magnesium, and potassium supplements. By applying this therapeutic regimen, a stable hypokalemia (at around 2 mmol/L) was established. During the following years, our patient experienced recurrent episodes of symptomatic hypokalemia, finally climaxing in severe respiratory muscle weakness with respiratory failure requiring temporary assisted ventilation. Only a detailed evaluation obtained by careful history taking revealed various interfering factors.

As an outpatient, he reported a few episodes of muscle weakness and gait disturbance which followed nights of moderately severe alcohol consumption. Alcohol is known to cause functional tubular defects, particularly with hypomagnesemia [9].

More severe episodes (more severe muscle cramping, attacks of pain and weakness) with more severe hypokalemia followed his consumption of large amounts of lemon juice. This presentation favors metabolic alkalosis and a further fall in plasma potassium concentration. Once the excessive lemon juice intake was discontinued, his potassium levels stabilized.

Another episode of even more pronounced symptomatic (muscle weakness extending from the legs to the upper extremities) hypokalemia (1.2 mmol/L) requiring admission to our hospital as an in-patient followed excessive consumption of sugar-containing iced tea. Consumption of large quantities of carbohydrates increases insulin secretion and thus enhances potassium influx into myocytes and hepatocytes, with a consequential fall in plasma potassium [10]. Additionally, his intake of low-sodium drinks was far from optimal to restore plasma volume and (presumably via hypovolemia-driven anti-diuretic hormone secretion) exacerbated his hyponatremia.

Last, the patient had progressive truncal weakness and finally presented with respiratory failure, necessitating temporary mechanical ventilation. At this point, extreme hypokalemia of 1.0 mmol/L was evident. The patient had started a new diet with restricted sodium intake. He later explained that he had vomited the day before being admitted to our hospital; in retrospect, the patient was found to have lost 2 kg of his body weight. As mentioned above, a low NaCl supply exaggerates volume depletion, enhancing compensatory secondary hyperaldosteronism and hypokalemia.

## Conclusion

The present case report illustrates the impressive complications encountered in a young patient with Gitelman's syndrome, especially in response to dietary challenges and excesses. It is remarkable that the patient's individual dietary preferences had a much larger impact on the symptoms attributable to his chronic disorder than any drug treatment prescribed by his physicians over the past four years. We have discussed some pathophysiological mechanisms leading to recurrent exacerbation of hypokalemia and clinical deterioration. Consequently, individual (mainly dietary) preferences must be monitored closely, and patients should be given dietary advice to avoid recurrent aggravation of hypokalemia with muscular weakness.

## Abbreviations

ACE: angiotensin-converting enzyme; ACTH: adrenocorticotropic hormone; ADH: anti-diuretic hormone; AT2R: angiotensin 2 receptor; Ca: calcium; ECG: electrocardiogram; HCO_3_: hydrogen carbonate; NaCl: sodium chloride; QTc: corrected QT interval; pCO_2_: partial pressure of carbon dioxide; pO_2_: partial pressure of oxygen; TSH: thyroid-stimulating hormone.

## Consent

Written informed consent was obtained from the patient for publication of this case report and any accompanying images. A copy of the written consent is available for review by the Editor-in-Chief of this journal.

## Competing interests

The authors declare that they have no competing interests.

## Authors' contributions

All authors read and approved the final manuscript.
